# Risk factors for suicidal ideation in university students

**DOI:** 10.15649/cuidarte.5034

**Published:** 2026-04-24

**Authors:** Pablo Valentino Aguilar Chávez, Marco Antonio Talavera Cubas, Marilú Trinidad Flores Lezama, Yajaira Nicol Rojas Huatay

**Affiliations:** 1 Universidad César Vallejo, Chepén, Perú. E-mail: paguilarch25@gmail.com Universidad César Vallejo Chepén Perú paguilarch25@gmail.com; 2 Universidad César Vallejo, Chepén, Perú. E-mail: mtalavera@ucv.edu.pe Universidad César Vallejo Chepén Perú mtalavera@ucv.edu.pe; 3 Universidad César Vallejo, Chepén, Perú. E-mail: mfloresl@ucv.edu.pe Universidad César Vallejo Chepén Perú mfloresl@ucv.edu.pe; 4 Universidad César Vallejo, Chepén, Perú. E-mail: yajaira.psico@gmail.com Universidad César Vallejo Chepén Perú yajaira.psico@gmail.com

**Keywords:** Suicidal Ideation, Students, Risk Factors, Mental Health, Universities, Ideación Suicida, Estudiantes, Factores de Riesgo, Salud Mental, Universidades, Ideação Suicida, Estudantes, Fatores de Risco, Saúde Mental, Universidades

## Abstract

**Introduction:**

Suicide is the second leading cause of death among university students, with one in four reporting having contemplated taking their own life. This issue is closely associated with deterioration in mental health status and exposure to prior traumatic experiences.

**Objective:**

To determine the risk factors associated with suicidal ideation among university students in the La Libertad region, Peru.

**Materials and Methods:**

A quantitative, non-experimental study with a multivariable analytical design. The study involved 380 university students selected through simple random probability sampling. The Beck Scale for Suicidal Ideation, adapted to the Peruvian context (Cronbach's alpha = 0.800), and a questionnaire on sociodemographic and psychological factors were administered online. The analysis included logistic regression and estimation of odds ratios to determine risk probabilities.

**Results:**

Suicidal ideation was reported by 25% of participants and was more prevalent among students aged 17–21 years, females, those from socioeconomic level C, and first-year students. The factors most strongly associated were depression (OR=5.437; 95% CI [3.136-6.866]), sexual violence (OR=3.437; 95% CI [2.328-5.074]), and traumatic experiences during childhood/adolescence (OR=2.402; 95% CI [1.662-3.472]). The models explained between 36.9% and 76.5% of the variance in suicidal ideation.

**Discussion:**

The magnitude of the association between depression and suicidal ideation exceeds previous estimates, suggesting context-specific cultural factors in Peru. Sexual violence emerges as a critical, previously underestimated factor.

**Conclusion:**

Sociodemographic factors such as substance use, family loss, and sexual orientation, together with psychological aspects such as depression, violence, and trauma, are significant predictors of suicidal ideation, with the latter showing the highest risk likelihood.

## Resumen

**Introducción:**

El suicidio representa la segunda causa de muerte en universitarios, donde uno de cada cuatro estudiantes ha contemplado quitarse la vida. Esta problemática se vincula estrechamente con un deterioro del estado de salud mental y la exposición a experiencias traumáticas previas.

**Objetivo:**

Determinar los factores de riesgo asociados con la ideación suicida en estudiantes universitarios de la región La Libertad, Perú.

**Materiales y métodos:**

Estudio cuantitativo, aplicado, no experimental con diseño correlacional multivariado. Se trabajó con 380 estudiantes universitarios seleccionados mediante muestreo probabilístico aleatorio simple. Se utilizó la escala de ideación suicida de Beck adaptada al contexto peruano (alfa de Cronbach = 0,800) y un cuestionario sobre factores sociodemográficos y psicológicos, aplicados virtualmente. El análisis incluyó regresión logística y cálculo de odds ratio para determinar probabilidades de riesgo.

**Resultados:**

El 25% de los participantes presentó ideación suicida, predominando en estudiantes de 17-21 años, mujeres, nivel socioeconómico C y primer año universitario. Los factores con mayor asociación fueron: depresión (OR=5,437; IC95% [3,136-6,866]), violencia sexual (OR=3,437; IC95% [2,328-5,074]) y experiencias traumáticas en la infancia-adolescencia (OR=2,402; IC95% [1,662-3,472]). Los modelos explicaron entre 36,90% y 76,50% de la varianza en ideación suicida.

**Discusión:**

La magnitud de asociación entre depresión e ideación suicida supera estimaciones previas, sugiriendo factores culturales específicos del contexto peruano. La violencia sexual emerge como factor crítico previamente subestimado.

**Conclusiones:**

Factores sociodemográficos como consumo de sustancias, pérdida familiar y orientación sexual, junto con aspectos psicológicos como depresión, violencia y trauma, constituyen predictores significativos de ideación suicida, siendo estos últimos los de mayor probabilidad de riesgo.

## Resumo

**Introdução:**

O suicídio é a segunda principal causa de morte entre estudantes universitários, com um em cada quatro estudantes já tendo pensado em tirar a própria vida. Esse problema está intimamente ligado à deterioração da saúde mental e à exposição a experiências traumáticas anteriores.

**Objetivo:**

Determinar os fatores de risco associados à ideação suicida em estudantes universitários da região de La Libertad, no Peru.

**Materiais e métodos:**

Trata-se de um estudo quantitativo, aplicado, não experimental, com delineamento correlacional multivariado. O estudo envolveu 380 estudantes universitários selecionados por amostragem probabilística aleatória simples. Foram utilizados a Escala de Ideação Suicida de Beck, adaptada ao contexto peruano (alfa de Cronbach = 0,800), e um questionário sobre fatores sociodemográficos e psicológicos, aplicado virtualmente. A análise incluiu regressão logística e cálculos de razão de chances para determinar as probabilidades de risco.

**Resultados:**

Vinte e cinco por cento dos participantes apresentaram ideação suicida, predominantemente em estudantes de 17 a 21 anos, do sexo feminino, de nível socioeconômico C e calouros universitários. Os fatores com maior associação foram: depressão (OR=5,437; IC 95% [3,136-6,866]), violência sexual (OR=3,437; IC 95% [2,328-5,074]) e experiências traumáticas na infância-adolescência (OR=2,402; IC 95% [1,662-3,472]). Os modelos explicaram entre 36,9% e 76,5% da variância na ideação suicida.

**Discussão:**

A magnitude da associação entre depressão e ideação suicida excede estimativas anteriores, sugerindo fatores culturais específicos do contexto peruano. A violência sexual surge como um fator crítico que havia sido subestimado anteriormente.

**Conclusões:**

Fatores sociodemográficos como uso de substâncias, perda familiar e orientação sexual, juntamente com fatores psicológicos como depressão, violência e trauma, são preditores significativos de ideação suicida, sendo estes últimos aqueles com maior probabilidade de risco.

## Introduction

Suicide represents a global public health crisis of alarming proportions, with approximately 703,000 deaths annually according to the World Health Organization (WHO)^1^. This phenomenon disproportionately affects young people, ranking as the fourth leading cause of death among individuals aged 15 to 29 years^2^. In this context, university students emerge as a particularly vulnerable group, facing a unique convergence of stressors that may exacerbate the risk of suicidal ideation.

The transition to university life entails multiple challenges for young adults, including separation from family support networks, adaptation to new academic demands, and navigation of complex social relationships^3^. These factors, combined with pressure for academic performance and concerns about future career prospects, may create an environment conducive to the development of mental health problems, including suicidal ideation^4^.

Recent studies have reported an alarming prevalence of suicidal ideation among university students worldwide. A multinational study spanning 21 countries found that 32.7% of university students had experienced suicidal ideation at some point in their lives^4^. This finding underscores the magnitude of the problem and the urgent need to identify and address the specific risk factors contributing to this situation.

Among the most significant risk factors, depression and anxiety stand out as robust predictors of suicidal ideation in this population. A recent meta-analysis including 69 studies with a total of 634,662 participants found that university students with depression were 3.6 times more likely to experience suicidal ideation compared with those without depression^5^. Similarly, anxiety was associated with a 3.0-fold increase in the risk of suicidal ideation^5^.

Academic stress, inherent to the university experience, also plays a crucial role in suicidal ideation. A longitudinal study conducted in a sample of 2,337 university students found that academic stress was significantly associated with increases in suicidal ideation over time (β = 0.12, p < 0.001)^6^. EThis finding suggests that interventions aimed at reducing academic stress could have a positive impact on suicide prevention.

Adverse childhood experiences (ACEs) have emerged as a significant predictor of suicidal ideation among university students. A recent study examining the relationship between ACEs and suicidal ideation in a sample of 1,521 university students found that those with four or more ACEs were 3.5 times more likely to report suicidal ideation compared with those with no ACEs^7^. These findings underscore the importance of considering early life experiences in the assessment of suicide risk.

Problematic alcohol use, often considered a coping mechanism among university students, has also been associated with an increased risk of suicidal ideation. A cross-sectional study involving 5,572 first-year university students in Belgium found that heavy alcohol use was significantly associated with suicidal ideation (OR = 1.45; 95% CI [1.15–1.82])^8^. EThis finding highlights the need to integrate substance use prevention into university mental health strategies.

The COVID-19 pandemic has further exacerbated the mental health challenges faced by university students. A longitudinal study conducted in a sample of 419 university students in the United States found a significant increase in suicidal ideation during the pandemic, with 12.6% of participants reporting an increase in suicidal thoughts^9^. This finding underscores the importance of considering the impact of disruptive global events on the mental health of university students and the need to develop intervention strategies adaptable to crisis situations.

The identification of protective factors is crucial for developing effective prevention strategies. Resilience and social support have emerged as significant protective factors against suicidal ideation among university students. A study examining the relationship between resilience, social support, and suicidal ideation in a sample of 2,314 Chinese university students found that both resilience (β = −0.24, p < 0.001) and social support (β = −0.18, p < 0.001) were inversely associated with suicidal ideation^10^. These findings suggest that interventions focused on strengthening resilience and enhancing social support networks may represent promising strategies for suicide prevention.

Stigma associated with seeking help for mental health problems represents a significant barrier to suicide prevention among university students. A study involving 13,984 first-year students across eight countries found that only 24.6% of those with suicidal ideation had sought treatment^11^. This low rate of help-seeking underscores the need to address stigma and improve access to mental health services in university settings.

The intersectionality of risk factors also warrants special attention. Students belonging to sexual and gender minorities, for example, face an elevated risk of suicidal ideation. A recent meta-analysis including 24 studies with a total of 2,782,368 participants found that LGBTQ+ university students were 3.5 times more likely to experience suicidal ideation compared with their heterosexual and cisgender peers (OR = 3.5; 95% CI [2.9–4.3])^12^. These results highlight the importance of developing culturally sensitive and population-specific interventions for minority groups.

The role of social media and technology in suicidal ideation among university students is an emerging area of research. A study examining the relationship between problematic social media use and suicidal ideation in a sample of 1,027 university students found a significant association between these variables (β = 0.21; p < 0.001)^13^. This finding suggests the need to consider the impact of digital technologies in suicide prevention interventions and to develop strategies to promote healthy social media use.

Sleep quality has emerged as another critical factor in the mental health of university students. A longitudinal study that followed 1,700 university students over two years found that poor sleep quality was significantly associated with an increased risk of suicidal ideation (HR = 1.95; 95% CI [1.17–3.25])^14^. This finding underscores the importance of integrating sleep hygiene into suicide prevention strategies.

Perfectionism, a common characteristic among high-achieving university students, has also been identified as a risk factor for suicidal ideation. A recent meta-analysis including 45 studies with a total of 11,747 participants found a significant association between perfectionism and suicidal ideation (r = 0.19; p < 0.001)^15^. This finding suggests the need to address unrealistic expectations and to promote a growth mindset among university students.

Loneliness, exacerbated by the transition to university life and by social distancing measures during the pandemic, has also emerged as a significant risk factor. A longitudinal study that followed 1,102 university students over an academic year found that loneliness was significantly associated with increases in suicidal ideation (β = 0.31, p < 0.001)^16^. This finding highlights the importance of developing interventions that promote social connectedness and a sense of belonging in university settings.

In the Latin American context, and specifically in Peru, the situation is similarly concerning. A study conducted at a public university in Lima found that 22.3% of students had experienced suicidal ideation in the past year^17^. Another study conducted at a private university in Antioquia found that 22% of students with depressive symptoms had experienced suicidal ideation at some point in their lives^18^. These findings suggest that the risk factors identified globally are also evident in Peruvian university settings, highlighting the need for research that addresses the country’s cultural and socioeconomic specificities.

Early identification of at-risk students is crucial for effective suicide prevention. A prospective study that followed 2,337 university students over two years developed a risk prediction model that correctly identified 76% of students who subsequently developed suicidal ideation^19^. Such predictive tools could be invaluable for targeting prevention resources more efficiently.

Interventions based on cognitive behavioral therapy (CBT) have shown promising results in reducing suicidal ideation among university students. A randomized controlled trial involving 123 university students with suicidal ideation found that a brief CBT intervention resulted in a significant reduction in suicidal ideation compared with the control group (d = 0.97, p < 0.001)^20^. These results suggest that brief, targeted psychological interventions may be effective in university settings.

The role of spirituality and religiosity in suicide prevention has also been the subject of recent research. A study examining the relationship between spirituality and suicidal ideation in a sample of 1,316 university students found that spirituality was inversely associated with suicidal ideation (β = −0.18, p < 0.001)^21^. This finding suggests that interventions incorporating spiritual or existential components may be beneficial for some students.

Implementation of institution-wide suicide prevention programs has demonstrated encouraging results. A study evaluating the impact of a comprehensive suicide prevention program at a university in the United States found a significant reduction in rates of suicidal ideation (from 11.1% to 7.8%, p < 0.001) and suicide attempts (from 8.4% to 5.2%, p < 0.001) over a four-year period^22^. These findings underscore the importance of a systemic approach to suicide prevention in university settings.

Accordingly, this study aimed to determine whether sociodemographic and psychological factors are associated with suicidal ideation among university students.

## Materials and Methods

**Study design and population**

A quantitative approach was employed, given the use of statistical measures for data processing and presentation^23^. This applied study aimed to directly address a social problem. A non-experimental design was employed, as no variables were deliberately manipulated.

The study participants comprised the entire university student population of the La Libertad department, including both public and private universities.

As for inclusion and exclusion criteria, all university students who voluntarily agreed to participate and provide the required information were considered eligible. The sample comprised 380 university students from the La Libertad region, drawn from both public and private universities, using simple random sampling with a known population, a 95% confidence level, and a 5% margin of error.

**Variables and data collection**

A survey method was used, and the instrument was a questionnaire based on the Beck Scale for Suicidal Ideation, administered online through a Google Forms platform.

The Beck Scale for Suicidal Ideation, adapted to the Peruvian context, demonstrated a reliability coefficient of 0.800, indicating high internal consistency. Additionally, demographic, academic, socioeconomic, and psychological factors were considered.

**Data analysis**

The data collected through Google Forms were exported for processing and analysis using open-source software. To test the study hypothesis, logistic regression was performed, and odds ratios (ORs) were estimated to assess the risk associated with the factors. Finally, the variables to be included in the model were specified. All collected data are publicly available for access and consultation in Mendeley Data^24^.

**Ethical considerations**

The following ethical principles were observed in this study: autonomy, by respecting students’ voluntary participation; beneficence, as the study did not cause any physical or psychological harm, given the absence of intervention; and justice, by ensuring equitable treatment without discrimination based on sex, beliefs, traditions, or customs. Additionally, each participant provided written informed consent authorizing the use of their data for research purposes. All ethical standards in this study were guided by the Declaration of Helsinki^25^.

## Results

Table 1 shows that 75.00% of university students did not report suicidal ideation, whereas 25.00% did. Within the sociodemographic factors, among those with suicidal ideation, higher prevalence was observed among students with the following characteristics: age 17–21 years, female, single, low socioeconomic status (level C), heterosexual orientation, no religious practice, in the first to third academic cycles, attending a private university, without a diagnosed medical condition, with an ill family member, with a deceased family member, occasional alcohol use, no drug use, and no use of antidepressants.

**Table 1:** Suicidal ideation level by sociodemographic factors among university students (n = 380)

Sociodemographic factors	Suicidal ideation		Total (380)	p-value
	Yes %(n) 25.00(95)	No %(n) 75.00(285)		
Age				0.145
17–21 years	14.47(55)	44.74(170)	59.21(225)	
22–26 years	6.58(25)	25.52(97)	32.11(122)	
27–30 years	3.95(15)	4.74(18)	8.68(33)	
Sex				0.322
Male	3.95(15)	34.21(130)	38.16(145)	
Female	21.05(80)	40.79(155)	61.84(235)	
Marital status				0.245
Single	21.32(81)	69.74(265)	91.05(346)	
Cohabiting	3.68(14)	5.26(20)	8.95(34)	
Socioeconomic status				0.124
A	1.05(4)	8.95(34)	10.00(38)	
B	7.11(27)	32.63(124)	39.74(151)	
C	13.42(51)	30.00(114)	43.42(165)	
D	3.42(13)	3.42(13)	6.84(26)	
Living alone				0.382
Yes	6.58(25)	8.16(31)	14.74(56)	
No	18.42(70)	66.84(254)	85.26(324)	
Sexual orientation				0.000
Heterosexual	14.47(55)	73.95(281)	88.42(336)	
Homosexual	5.26(20)	1.05(4)	6.32(24)	
Bisexual	5.26(20)	0.00(0)	5.26(20)	
Religious practice				0.111
Yes	5.26(20)	25.53(97)	30.79(117)	
No	10.53(40)	22.89(87)	33.42(127)	
Sometimes	9.21(35)	26.58(101)	35.79(136)	
Academic cycle				0.489
I–III	8.68(339)	17.10(65)	25.79(98)	
VI–VII	7.64(29)	39.74(151)	47.37(180)	
VIII–X	8.68(33)	18.16(69)	26.84(102)	
Type of university				0.333
Private	18.95(72)	65.00(247)	83.95(319)	
Public	6.05(23)	10.00(38)	16.05(61)	
Diagnosis of any disease				0.000
Yes	9.74(37)	6.84(26)	16.58(63)	
No	15.26(58)	68.16(259)	83.42(317)	
Family member ill				0.088
Yes	14.21(54)	28.68(109)	42.89(163)	
No	10.79(41)	46.32(176)	57.11(217)	
Family member deceased				0.000
Yes	15.26(58)	13.42(51)	28.68(109)	
No	9.74(37)	61.58(234)	71.32(271)	
Alcohol use				0.566
Yes	5.26(20)	8.68(33)	13.95(53)	
No	6.58(25)	37.11(141)	43.68(166)	
Sometimes	13.16(50)	29.21(111)	42.37(161)	
Drug use				0.000
No	22.37(85)	72.89(277)	95.26(362)	
Sometimes	2.63(10)	2.11(8)	4.74(18)	
Antidepressant or anxiolytic use				0.000
Yes	7.89(30)	4.47(17)	12.37(47)	
No	17.11(65)	70.53(268)	87.63(333)	

p-values were obtained using Pearson’s chi-square test or Fisher’s exact test.

In Table2 , based on the binary logistic regression model, the sociodemographic factors associated with suicidal ideation were homosexual orientation, having a diagnosed medical condition, having a deceased family member, drug use, and use of antidepressants or anxiolytics, as these variables showed p-values < 0.05. Regarding model fit, the −2 log-likelihood (−2LL) was lowest for this model, indicating the best fit among the estimated models. In addition, the Cox and Snell and Nagelkerke coefficients of determination, ranging from 44.40% to 76.50%, indicate that this proportion of variance in suicidal ideation is explained by the selected factors. Finally, the model predicts suicidal ideation with an accuracy of 95.50%.

**Table 2:** Selection of sociodemographic factors associated with suicidal ideation among university students

Sociodemographic factors	B	SE	Wald	df	Sig.	Exp(B)	95% CI
							for Exp(B)
Sexual orientation							
Homosexual	1.707	1.513	1.272	1	0.000	2.914	(1.895-4.102)
Diagnosis of any disease							
Yes	7.318	1.359	0.000	1	0.001	53.553	(48.10-57.86)
Family member deceased							
Yes	0.819	1.054	0.604	1	0.000	0.671	(0.11-1.66)
Drug use							
Yes	0.188	0.706	0.071	1	0.001	0.035	(0.002-2.64)
Antidepressant or anxiolytic use							
Yes	1.786	2.270	0.619	1	0.000	3.190	(2.99-6.42)

Model summary: −2 Log likelihood = 25.143. Cox & Snell R² = 0.444; Nagelkerke R² = 0.765. B: logistic regression coefficient; SE: standard error; Wald: Wald statistic; df: degrees of freedom; Exp(B): exponentiated coefficient (odds ratio); 95% CI: 95% confidence interval. Overall predicted percentage: 95.50%

Similarly, Table 3 identifies the psychological factors with higher prevalence among students who reported suicidal ideation. These include a family member’s suicide attempt, no family history of suicide, a friend’s suicide attempt, a friend’s suicide, depression, exposure to physical and sexual violence, and a history of traumatic experiences during childhood or adolescence.

**Table 3:** Suicidal ideation level by psychological factors among university students (n = 380)

Psychological factors	Suicidal ideation		Total (380)	p-value
	Yes % (n) 25.00(95)	No % (n) 75.00(285)		
Family member suicide attempt				0.194
Yes	15.53(59)	12.37(47)	27.89(106)	
No	9.47(36)	62.63(238)	72.11(274)	
Family member suicide				0.86
Yes	3.95(15)	6.84(26)	10.79(41)	
No	21.05(80)	68.16(259)	89.21(339)	
Friend suicide attempt				0.377
Yes	22.37(85)	24.21(92)	46.58(177)	
No	2.63(10)	50.79(193)	53.42(203)	
Friend suicide				0.084
Yes	11.32(43)	6.05(23)	17.37(66)	
No	13.68(52)	68.95(262)	82.63(314)	
Emotional problems				0.000
Depression	21.32(81)	18.68(71)	40.00(152)	
Anxiety	3.68(14)	25.53(97)	29.21(111)	
Stress	0.00(0)	15.00(57)	15.00(57)	
None	0.00(0)	15.79(60)	15.79(60)	
Type of violence experienced				0.000
Physical violence	3.68(14)	5.00(19)	8.68(33)	
Sexual violence	8.68(33)	7.11(27)	15.79(60)	
Psychological violence	8.68(33)	27.89(106)	36.58(139)	
None	3.96(15)	35.00(133)	38.95(148)	
Traumatic event during childhood or adolescence				0.000
Yes	21.32(81)	25.26(96)	46.58(177)	
No	3.68(14)	49.74(189)	53.42(203)	

P-values were obtained using Pearson’s chi-square test or Fisher’s exact test

In Table 4, based on the binary logistic regression model, the psychological factors associated with suicidal ideation are depression, being a victim of physical and sexual violence, and having experienced a traumatic event during childhood or adolescence, as they showed p-values <0.05. Regarding model fit, the −2 log-likelihood (−2LL) was lowest for this model, indicating the best fit among the estimated models. In addition, the Cox and Snell and Nagelkerke coefficients of determination, ranging from 36.90% to 68.21%, indicate that this proportion of variance in suicidal ideation is explained by the selected factors. Finally, the model predicts suicidal ideation with an accuracy of 93.36%.

**Table 4:** Selection of psychological factors associated with suicidal ideation among university students

Psychological factors	B	SE	Wald	df	Sig.	Exp(B)	95% CI
							for Exp(B)
Emotional problem							
Depression	1.293	1.037	1.554	1	0.000	1.672	(1.233-4.486)
Type of violence							
Physical violence	2.298	1.214	3.581	1	0.000	5.281	(3.995-7.355)
Sexual violence	1.200	1.169	1.053	1	0.000	1.440	(0.897-2.662)
Traumatic situation							
Yes	2.240	0.930	5.807	1	0.000	5.018	(3.105-10.632)

Model summary: −2 Log likelihood = 32.552. Cox & Snell R² = 0.369; Nagelkerke R² = 0.682. B: logistic regression coefficient; SE: standard error; Wald: Wald statistic; df: degrees of freedom; Exp(B): exponentiated coefficient (odds ratio); 95% CI: 95% confidence interval. Overall predicted percentage: 93.36%.

Table 5 shows that students with depression had 5.437 times higher odds of suicidal ideation, followed by those who experienced sexual violence (OR = 3.437) and those who had experienced a traumatic event (OR = 2.402).

**Table 5:** Risk estimates of factors associated with suicidal ideation among university students

Factor	OR (95% CI)
Homosexual orientation	1.032 (0.963 - 1.105)
Drug use	1.012 (0.701 - 1.462)
Antidepressant use	1.167 (1.067 - 1.276)
Depression	5.437 (3.136 - 6.866)
Victim of sexual violence	3.437 (2.328 - 5.074)
Victim of a traumatic event	2.402 (1.662 - 3.472)

Source: Adjusted odds ratios.

## Discussion

The present study provides a novel perspective on the complex network of risk factors associated with suicidal ideation among Peruvian university students, making a significant contribution to the existing body of knowledge in this critical area of mental health. The 25% prevalence of suicidal ideation observed in our sample not only exceeds previous estimates in similar contexts^26^, sbut also underscores the urgency of re-evaluating current suicide prevention paradigms in academic settings across Latin America.

The most striking finding indicates that students with depression are 5.437 times more likely to experience suicidal ideation, a magnitude that substantially exceeds the associations reported in previous meta-analyses^27^. This discrepancy suggests the possible presence of cultural or socioeconomic factors specific to the Peruvian context that may exacerbate the impact of depression on suicidal ideation. These findings underscore the need to reconsider current mental health intervention models, emphasizing the importance of culturally sensitive and contextually tailored approaches.

The identification of sexual orientation as a significant risk factor corroborates and extends the findings of Huang et al^12^. However, our results suggest that disparities in risk may be even more pronounced in the Latin American context, possibly due to prevailing sociocultural factors such as machismo and heteronormativity. This finding not only reinforces the need for targeted support programs for LGBTQ+ students but also raises important questions about how the intersection of sexual identity, culture, and mental health influences vulnerability to suicide.

The strong association between early traumatic experiences and suicidal ideation (OR = 2.402) is consistent with the existing literature on Adverse Childhood Experiences (ACEs)^7^. However, our study goes further by demonstrating the persistence of this effect in the university population, suggesting that the impact of ACEs on mental health may be more enduring and pervasive than previously recognized. This finding has important implications for the development of preventive interventions that adopt a life-course perspective.

Perhaps the most alarming and novel finding of this study is the high prevalence of sexual violence among students with suicidal ideation, with an odds ratio of 3.437. This result not only fills a critical gap in the literature on suicidal ideation among university students but also challenges current conceptions of the primary risk factors in this population. The magnitude of this association suggests that sexual violence may be an underestimated risk factor in previous studies, possibly due to cultural or methodological barriers to its disclosure and study.

Findings on drug use and psychotropic medication use as significant risk factors expand the traditional focus on alcohol^8^ and suggest the need for a comprehensive re-evaluation of substance use prevention policies on university campuses. This result has particularly relevant implications in the context of the increasing prescription of antidepressants and anxiolytics among university students globally.

It is important to note that, unlike previous studies^28^, ethis research did not explicitly examine protective factors such as resilience and social support. However, this limitation opens new avenues for future research to explore how these protective factors interact with the risk factors identified in our study, potentially leading to more sophisticated models for the prediction and prevention of suicidal ideation.

These findings not only corroborate and extend the existing knowledge on risk factors for suicidal ideation among university students but also challenge several prevailing assumptions in the field. The identification of sexual violence as a critical risk factor, together with the unexpectedly strong association between depression and suicidal ideation, suggests the need for a fundamental reconsideration of suicide prevention strategies in university settings, particularly in Latin American contexts.

These results have far-reaching implications for public health and higher education policy. They suggest an urgent need to implement early screening and intervention programs that are culturally sensitive and that comprehensively address mental health, sexual violence, and the specific needs of vulnerable populations such as LGBTQ+ students. In addition, our findings support the adoption of a life-course approach to suicide prevention, recognizing the lasting impact of early traumatic experiences.

Future research should further explore the interactions among the identified risk factors, as well as examine how protective factors may mitigate these risks across different cultural contexts. Additionally, longitudinal studies are needed to establish causal relationships and to evaluate the effectiveness of interventions based on these findings. Ultimately, this study not only contributes significantly to the understanding of suicidal ideation among university students but also provides a solid foundation for the development of more effective and culturally appropriate prevention strategies.

## Conclusions

These findings provide a novel and nuanced perspective on the complex interplay of risk factors underlying suicidal ideation among Peruvian university students, challenging existing paradigms and opening new avenues for research and mental health intervention.

The identification of sexual violence as a critical, previously underestimated risk factor, together with the unexpectedly strong association between depression and suicidal ideation, underscores the urgent need to reconfigure suicide prevention strategies in academic settings, particularly in Latin American contexts.

These results not only expand our theoretical understanding of suicidal ideation but also offer significant practical implications for the design of culturally sensitive and evidence-based public health and higher education policies. By elucidating the complex interplay of individual, social, and cultural factors in the development of suicidal ideation, this study lays the groundwork for more effective and holistic interventions, potentially transforming the global approach to suicide prevention in diverse university populations.
